# Hyoid Bone and Thyroid Cartilage Metastases from Sigmoid Colon Adenocarcinoma: A Case Report

**DOI:** 10.4274/balkanmedj.2015.1817

**Published:** 2017-05-15

**Authors:** Djurdja Bracanovic, Vesna Vukovic, Aleksa Janovic, Davorin Radosavljevic, Zoran Rakocevic

**Affiliations:** 1 Department of Radiology, Belgrade University School of Dentistry, Belgrade, Serbia; 2 Clinic of Diagnostic Imaging, Serbia Radiology and Oncology Institute, Belgrade, Serbia; 3 Clinic for Medical Oncology, Serbia Radiology and Oncology Institute, Belgrade, Serbia

**Keywords:** Hyoid bone, thyroid cartilage, adenocarcinoma metastases, computerized tomography, bone scintigraphy

## Abstract

**Background:**

Secondary tumours of the hyoid bone and thyroid cartilage are extremely rare. In this paper, we present a case of the hyoid bone and thyroid cartilage metastases in a patient treated for sigmoid colon adenocarcinoma.

**Case Report:**

Four years after sigmoid colon adenocarcinoma was diagnosed and treated with surgery and chemotherapy, the patient developed bone metastases in the left sacroiliac joint and right proximal humerus. Although the patient did not complain of any related symptoms, in a bone scintigraphy the accumulation of Technetium-99m was incidentally detected in the two sites of the anterior neck. On ultrasound examination there were two hyperechoic and heterogeneous masses with calcifications placed in front of the hyoid bone and thyroid cartilage. Computerized tomography demonstrated massive hyoid bone and thyroid cartilage destruction.

**Conclusion:**

In patients with progressive sigmoid colon adenocarcinoma, destruction of the hyoid bone and thyroid cartilage could be suspected for metastases.

Secondary tumours of the hyoid bone and thyroid cartilage are extremely rare. Primary malignancies that frequently metastasize to the hyoid bone and/or the thyroid cartilage arise from the adjacent tissues, e.g. the larynx, the vallecula and pyriform sinus (1). Only a few cases of the hyoid bone metastasis from distant primary malignancies, such as renal cell carcinoma and hepatocellular carcinoma, had been reported to date (2). Similarly, metastases to the thyroid cartilage were occasionally described in patients with hepatocellular carcinoma and lung adenocarcinoma (3,4). In this paper, we present a rare case of the hyoid bone and thyroid cartilage metastases in a patient treated for sigmoid colon adenocarcinoma.

## CASE PRESENTATION

A 63-year-old male patient was admitted to the hospital due to repeated episodes of abdominal pain and rectal bleeding. Initial workup included colonoscopy, which revealed an ulcerated mass in the sigmoid colon. Results of the biopsy were consistent with an adenocarcinoma. Serum levels of carcinoembryonic antigen (CEA) (89 ng/mL) and cancer antigen 19-9 (CA 19-9) (326.7 U/mL) were elevated. Contrast-enhanced computerized tomography (CT) (Siemens Somatom Sensation 16, Munich, Germany) of the abdomen and pelvis showed presence of mesenteric lymphadenopathies and hypodense lesions in liver segments III, IV and V. The lesions in the liver were highly suggestive for metastases. According to the tumour board recommendation, a left hemicolectomy with regional lymph node removal was performed and the diagnosis of an invasive adenocarcinoma was confirmed [Dukes classification C; T3c N2 (6/9) L1 V1 R0]. The treatment was continued with four cycles of bevacizumab plus oxaliplatin-based chemotherapy, after which the patient underwent hepatic segmentectomy. Three months later, the chemotherapy was modified to FOLFOX regimen (Leucovorin Calcium, Fluorouracil, Oxaliplatin; 12 cycles) due to a CT-verified recurrence of the liver metastases. After a follow-up of 3 years, the chemotherapy regimen was changed to FOLFIRI (Leucovorin Calcium, Fluorouracil, Irinotecan Hydrochloride). Due to slow, continuous progression of the liver metastases, the chemotherapy protocol was changed to FOLFOX and XELOX (Capecitabine, Oxaliplatin) regimen. In this period, genetic analysis revealed a mutation of Gly12Asp in 12 codon of K-Ras gene.

**Figure 1:**
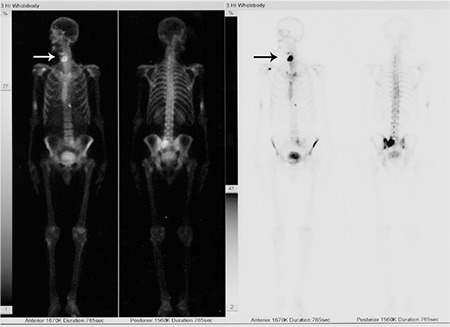
Bone scintigraphy shows accumulation of Tc-99m at the two sites of the anterior neck (arrows). Note also the accumulation of Tc-99m in the left iliac bone, sacrum and the right proximal humerus.

**Figure 2:**
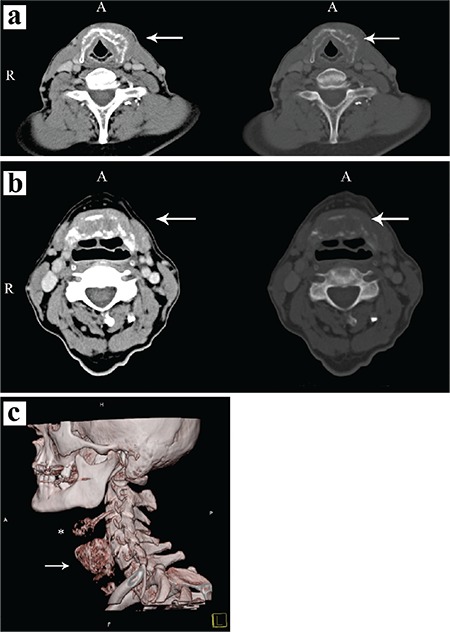
Contrast-enhanced CT scans of the neck: (a) Enlargement and osteolyses of the hyoid bone (arrows) presented in a soft tissue and bone window (A-anterior, R-right); (b) Tumour mass in the thyroid cartilage with reactive sclerosis (arrows) presented in a soft tissue and bone window (A-anterior, R-right); (c) 3D reconstruction image of thyroid cartilage (arrow) and hyoid bone (asterisk) defects (A-anterior, H-head, P-posterior, F-foot).

After the administration of the last chemotherapy cycle, the patient started to complain of severe pain in the left gluteal region and right arm. The significant elevation of serum levels of CEA (60 ng/mL) and CA 19-9 (255.5 U/mL) were detected. Osteolytic lesions within the left half of the sacrum and adjacent part of the iliac bone observed during CT examination indicated the presence of skeletal metastases and the patient underwent a bone scintigraphy. In addition to the increased accumulation of Technetium-99m in the left iliac bone, sacrum and the right proximal humerus, the radiopharmaceutical also accumulated at two sites in the anterior neck (Figure 1). Physical examination of the neck revealed two small, painless, palpable masses located anteriorly at the level of the hyoid bone and superior to the left lobe of the thyroid gland. When asked, the patient reported no significant symptoms. Ultrasound examination revealed a heterogeneous solid mass, with multiple small hyperechoic foci showing acustic shadowing and increased vascularization on colour Doppler. There was no cervical lymphadenopathy and the thyroid gland appeared normal. CT of the neck demonstrated enlargement of the body of the hyoid bone with massive osteolysis and cortical destruction (Figure 2a, 2c). The mass from the hyoid bone showed slight soft tissue extension toward the region of the vallecula and the epiglottis. Enlargement and destruction of the thyroid cartilage, predominantly of its left lamina, was also noted (Figure 2a,2b,2c). The airway lumen was not affected. There were no pathological changes in the adjacent structures of the neck. At this stage the patient’s performance status (PS) was scored as PS3. The multidisciplinary tumour board (MTB) proposed fine needle aspiration of the mass in the hyoid bone. Even though the patient was fully informed about the suggested procedure, he still refused it. The MTB did not insist on performing the biopsy, bearing in mind that histopathological verification in this case would not influence the decision regarding choice of therapy. According to the MTB recommendation, the patient underwent palliative radiotherapy for the metastases in the sacrum.

An informed consent was obtained from the patient.

## DISCUSSION

Gastrointestinal adenocarcinoma is among the most common malignancies worldwide and one of the most common causes of cancer-related death. Although it commonly spreads to the regional lymph nodes, liver and lungs, it may also metastasize to unusual sites, such as paranasal sinuses and cryptorchid testis (5). Its metastases to the larynx and adjacent structures have been only occasionally reported (6).

In the patient described in this case, the hyoid bone and thyroid cartilage lesions were detected simultaneously with osteolytic lesions in other skeletal sites. In the context of differential diagnosis we have considered diseases that may cause osteolytic lesions in the hyoid bone (i.e. primary bone tumours, multiple myeloma, secondary tumours from different primary malignancies). However, considering the status of primary malignant metastatic disease and elevated serum levels of tumour markers, the lesions in the hyoid bone and thyroid cartilage were highly suggestive of metastatic involvement by adenocarcinoma. This diagnosis was supported by the fact that unusual skeletal metastases of gastrointestinal adenocarcinoma more frequently occur in patients with disseminated disease than in patients without liver and/or lung involvement (7,8). Moreover, sigmoid tumours had the highest rate of bone metastasis in long-term follow-up in comparison to the right and left colon (8).

Although hyoid bone and thyroid cartilage involvement by sigmoid colon adenocarcinoma was highly suggestive, a biopsy was considered. However, the appropriateness of this procedure was brought into question. At the time of the bone metastases diagnosis, the patient had already developed multiple metastases to the liver and other skeletal sites. Severe pain due to the skeletal metastases contributed negatively to the patient’s general condition (low PS). Having in mind that K-Ras mutation is a predictive factor of survival in patients with colorectal cancer, this also contributed to the poor prognosis of the patient (9). In patients in a similar condition, palliative therapy had been suggested as a treatment of choice in order to improve the quality of life (6). Bearing in mind that the patient refused the biopsy as well as that the histological verification of masses in the neck would not influence the tumour board decision on the therapy, insisting on the procedure seemed unjustified.

Metastases of the colorectal adenocarcinoma in this case probably involved the hyoid bone haematogenously. The spread along the branches of the superior laryngeal artery was previously suggested as the most likely pathway (10). Given that branches of the lingual artery also supply the hyoid bone, spreading through these vessels might also be possible.

In summary, destruction of the hyoid bone and thyroid cartilage should be suspected for metastases in patients with progressive sigmoid colon adenocarcinoma. We suggest bone scintigraphy accompanied by CT examination in order to detect silent bone metastases.
